# Detection of Pulmonary Embolism with Gallium-68 Macroaggregated Albumin Perfusion PET/CT: An Experimental Study in Rabbits

**DOI:** 10.1155/2020/5607951

**Published:** 2020-06-29

**Authors:** Aziz Gültekin, Mustafa Çağdaş Çayır, Ayşe Uğur, Ferda Bir, Doğangün Yüksel

**Affiliations:** ^1^Pamukkale University Medical Faculty, Department of Nuclear Medicine, Denizli, Turkey; ^2^Pamukkale University Medical Faculty, Department of Cardiovascular Surgery, Denizli, Turkey; ^3^Pamukkale University Education and Research Hospital, Department of Nuclear Medicine, Denizli, Turkey; ^4^Pamukkale University Medical Faculty, Department of Pathology, Denizli, Turkey

## Abstract

This study was designed to evaluate the accuracy of detecting pulmonary embolism (PE) using the Technegas SPECT/CT combined with ^68^Ga PET/CT in a rabbit model. One hour after artificial PE (*n* = 6) and sham (*n* = 6) models were created, Technegas SPECT/CT ventilation and ^68^Ga-MAA PET/CT perfusion scan (V/Q scan) were performed. Ventilation imaging was performed first on all cases. Technegas SPECT/CT and ^68^Ga-MAA PET/CT images were evaluated by a nuclear medicine physician who recorded the presence, number, and location of PE on a per-lobe basis. The sensitivity, specificity, and accuracy of Technegas SPECT/CT and ^68^Ga-MAA PET/CT for detecting PE were calculated using a histopathological evaluation as a reference standard. A total of 60 lung lobes were evaluated in 12 rabbits, and PE was detected in 20 lobes in V/Q scans and histopathological analysis. The overall sensitivity, specificity, and accuracy were 100%, 100%, and 100%, respectively, for both the Technegas SPECT/CT and ^68^Ga-MAA PET/CT V/Q scans. Technegas/^68^Ga-MAA V/Q scans have good sensitivity, specificity, and accuracy in the detection of PE in this animal model study.

## 1. Introduction

Pulmonary embolism is fatal [1]. The mortality of untreated PE is approximately 25–30%, and in treated patients, it has been reported to be less than 10% [1–3]. The high mortality rate for PE requires extreme alertness in diagnosis [4]. The most commonly used imaging methods for the diagnosis of PE are V/Q-SPECT and computed tomography pulmonary angiography (CTPA). Although these tests have been widely validated, they have some limitations [5]. An important limitation of V/Q-SPECT in the recent years is the difficulty in obtaining ^99m^Tc worldwide. ^99m^Tc is obtained from a ^99^Mo/^99m^Tc generator. ^99m^Tc crises occasionally occur in the world due to shortages in the supply of ^99^Mo. Therefore, there is a search for new alternative radionuclides to ^99m^Tc [6, 7]. With the spread of ^68^Ge/^68^Ga generators, new searches in nuclear medicine and molecular imaging have emerged for PE. For PET studies, V/Q radiopharmaceuticals (RFs) labelled with ^68^Ga are relatively easy to produce, offering higher spatial resolution than SPECT studies [8, 9]. There are a limited number of preclinical and clinical lung perfusion studies with ^68^Ga-MAA perfusion PET/CT [10].

The aim of our preclinical study was to evaluate the diagnostic accuracy of combined ^68^Ga-MAA perfusion PET/CT and Technegas ventilation SPECT/CT to detect PE in a rabbit model.

## 2. Materials and Methods

### 2.1. ^68^Ga-MAA Labelling and Quality Control

A volume of 7 mL ^68^GaCl_3_ eluted with 0.1 N HCl (from ABX D-01454 Radeberg, Germany) from a ^68^Ge/^68^Ga generator was passed through a PSH + cartridge to yield ^68^Ga of high purity. ^68^Ga-chloride was added to 1.5 M 4-(2-hydroxyethyl) piperazine-1-82 ethanesulfonic acid (HEPES) buffer solution (from ABX D-01454), bringing the pH to 4–5. Since the prewash step was important before adding ^68^Ga to MAA particles in commercial kits, we conducted the labelling process by washing with saline the MAA particles in our study. After vigorous mixing with MAA (inside 5 mL sterile saline), the ^68^Ga-MAA suspension was incubated in a heat block at 90°C (7 min). The pH value of the ^68^Ga-MAA solution in the product vial was evaluated, and a 5 *μ*L sample was titrated onto Whatman No. 40 paper (8 cm long, 1 cm thick) and passed through a mobile phase to calculate the RF values. Two different solutions were used as the mobile phase: methanol/ammonium acetate 10% (1 : 1) and acetone/glacial acetic acid (9 : 1). Radioactivity measurements were performed with a dose calibrator. The structure, morphology, and chemical composition of ^68^Ga-MAA were analyzed by scanning electron microscopy (SEM)/energy dispersive X-ray (EDX). The resulting ^68^Ga-labeled MAA particles were examined for structural degradation.

### 2.2. Animal Experiments

**Figure 1:**
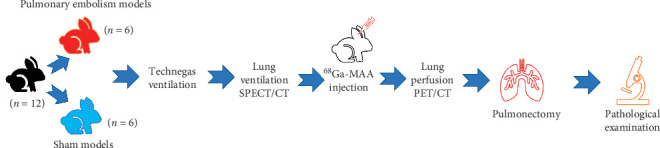
Flowchart of the rabbit experiment.

The flowchart of the rabbit experiment is shown in Figure 1. This preclinical study was conducted following ethical approval from the Animal Experiments Ethics Committee (Jan 11, 2019; 60758568-020/2716). The European Union directives were followed. The gender of the animal was not considered to be a factor in the experimental design. All rabbits were cared for and kept in separate cages with 12 h day and night cycles at 25°C during all procedures and fed ad libitum. Twelve New Zealand rabbits (*Oryctolagus cuniculus*) that weighed 2350–3000 g were divided into 2 groups: PE (*n* = 6) and sham (*n* = 6). In all rabbits, ketamine 35 mg/kg (Ketasol 10 mL, Richter Pharma, Austria) and xylazine 5 mg/kg (Rompun 25 ml, Bayer, USA) were injected intramuscularly to induce general anaesthesia. After shaving the right femoral region of the rabbits, the skin was disinfected. Pulmonary embolism was induced in 6 rabbits via gelatin sponge (GelSpon, Eucare Pharmaceuticals Ltd, Chennai, India) plugs through the right femoral vein. For 6 rabbits, 5 mL of saline was injected into the right femoral vein, and these animals were included in the sham group. The right femoral vein was surgically exposed. An 18 gauge (green) Angiocath (Gloflon, Global Medikit Ltd, Delhi, India) was placed. From a 80 × 50 × 10 mm gelatin sponge, four 2 × 2 × 10 mm gelatin sponges per rabbit were cut under sterile conditions and softened in 5 cc saline. The first six rabbits (PE group) were injected with four 2 × 2 × 10 mm gelatin sponge plugs as by Yang et al. [11]. The remaining 6 rabbits (sham group) were only injected with 5 mL of saline.

### 2.3. Ventilation SPECT/CT with Technegas

Technegas was produced from a Technegas generator according to the manufacturer's instructions. After the rabbits were anesthetized, the Technegas was ventilated with the aid of a mouth mask modified from a baby mask. Technegas was passively ventilated to rabbits with the help of an AMBU on the device. After a ventilation period of approximately one minute, imaging was performed in the prone position.

SPECT/CT imaging was conducted using a SPECT/CT device equipped with a dual-head hybrid gamma camera and combined within the same gantry with a flat-panel CT with a low-dose X-ray tube (2.5 mA, 120 kVp; Philips Brightview XBT; Cleveland, OH, USA). SPECT/CT images of the thorax were obtained immediately after Technegas ventilation. CT images were generated using a 512 × 512 matrix with a slice thickness of 1 mm. The SPECT images were captured using a low-energy high-resolution collimator (140 keV photoelectric pic, 20% energy window). They were acquired using a 64 × 64 matrix (64 frames; 30 s per frame). Images in each plane were captured by rotating 360 degrees around the animals. The images were reconstructed using an Astonish filter and the filtered backprojection method (cutoff, 3; order, 8) (AutoSPECTPro; Philips Intellispace Portal). Attenuation correction was performed using transverse, sagittal, and coronal CT imaging. The SPECT and CT images were fused to generate SPECT/CT images.

### 2.4. ^68^Ga-MAA PET/CT Perfusion Imaging

Immediately after the ventilation study was completed, ^68^Ga-MAA PET/CT perfusion imaging was performed. The rabbits were anesthetized. Immediately after injection of 37 MBq ^68^Ga-MAA into the ear marginal vein, imaging was performed in the prone position. The rabbits were examined using a PET/CT scanner (Gemini TF TOF PET-CT; Philips, Cleveland, OH; 3D mode, slice thickness of 5 mm, 4 × 4 × 22 mm, 256 × 256 matrix, transverse FOV 576 mm, and axial FOV 180 mm). Whole body emission scans were acquired for 2 minutes per position without intravenous contrast injection. Transmission images were obtained by low-dose CT (90 mA, 100 kV, 16 CT detectors, slice thickness of 5 mm). Attenuation correction was performed for PET images using CT maps. The ordered subsets-expectation maximization algorithm included 33 subsets and 3 iterations. PET images were reconstructed. Transverse, sagittal, and coronal sections (5 mm thickness) were created from PET/CT fusion images and evaluated using the Philips Fusion Viewer software (ver. 2.1; Philips Healthcare, Best, the Netherlands).

### 2.5. V/Q Scan Evaluation for Pulmonary Emboli

For each rabbit, the pulmonary perfusion PET/CT and ventilation SPECT/CT findings were assessed for each of the five lung lobes (right upper, middle, and lower lobes and left upper and lower lobes). Furthermore, ^68^Ga-MAA PET/CT images were blindly evaluated by a nuclear medicine physician. The nuclear medicine physician marked the perfusion and ventilation defects in a chart showing lung lobes for each rabbit. The EANM guidelines for ventilation/perfusion SPECT were used to diagnose emboli [12, 13]. The presence of PE was defined as one or more wedge-shaped mismatched V/Q defects.

### 2.6. Histopathologic Examination

Pathological evaluation was accepted as the reference standard for this study. Rabbits were sacrificed with high-dose anaesthesia after lung V/Q imaging. The lungs and heart were removed in blocks. The materials were fixed with formaldehyde. Pathological slices were obtained from each lung lobe from the hilus to the periphery at 1-mm intervals. The sections were stained with hematoxylin and eosin. Pulmonary emboli were diagnosed when gelatin sponges were stained with HE in the pulmonary arteries.

### 2.7. Statistical Analysis

Data were analysed with the SPSS 24.0 package software (IBM, Armonk, NY, USA). Continuous variables are presented as the mean ± standard deviation, whereas categorical variables are provided as frequencies and percentages. Pathological results were accepted as the reference. Sensitivity, specificity, and accuracy values of combined ^68^Ga-MAA PET/CT perfusion and Technegas SPECT/CT ventilation imaging for PE were calculated.

## 3. Results

**Figure 2:**
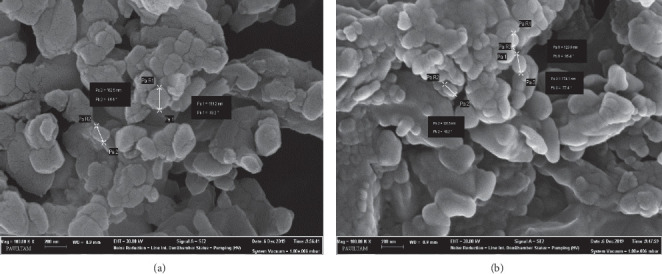
SEM images of the MAA particles (a) before and (b) after labelling with ^68^Ga (200 nm, Mag ×100.00 K, EHT 30.00 kV).

We found that 99% radiochemical purity of ^68^Ga-MAA was obtained by washing with saline. ^68^Ga-MAA labelled at an optimized pH (pH 4-5) was available for clinical application at 80% total activity 15 minutes after labelling. No radical scavenger was added during the labelling to increase the synthesis yield. The labelling procedure we developed, provided maximum ^68^Ga-MAA activity. SEM analysis showed the ^68^Ga-MAA particles to remain within their original size range (Figure 2).

The ^68^Ga-MAA PET/CT perfusion, Technegas SPECT/CT ventilation imaging, and histopathological results of all rabbits are shown in Table 1. In terms of PE diagnosis, V/Q scan findings and HP findings are fully compatible on a case-by-case basis (100%). When evaluating the 6 rabbits (PE models) and 30 lung lobes separately, lobar and segmental perfusion defects were detected in 20 lobes. Ventilation was normal in all 30 lobes. Perfusion and ventilation defects were not detected in any of the 30 lung lobes of the 6 rabbits in the sham group.

V/Q scan findings and HP findings were compared at the lobar levels. Since the rabbit has a small lung, it was not possible to evaluate at the segmental/subsegmental level in V/Q scan and HP examination. Lung V/Q scan and HP findings were fully compatible (Table 2).

**Figure 3:**
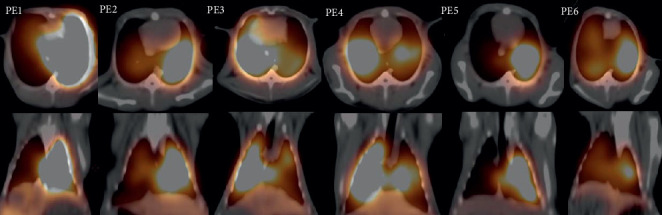
^68^Ga-MAA perfusion PET/CT images of all PE rabbits. The images in the first row are transaxial sections, and the images in the second row are coronal sections. Multiple wedge-shaped perfusion defects were observed in the images for all rabbits.

**Figure 4:**
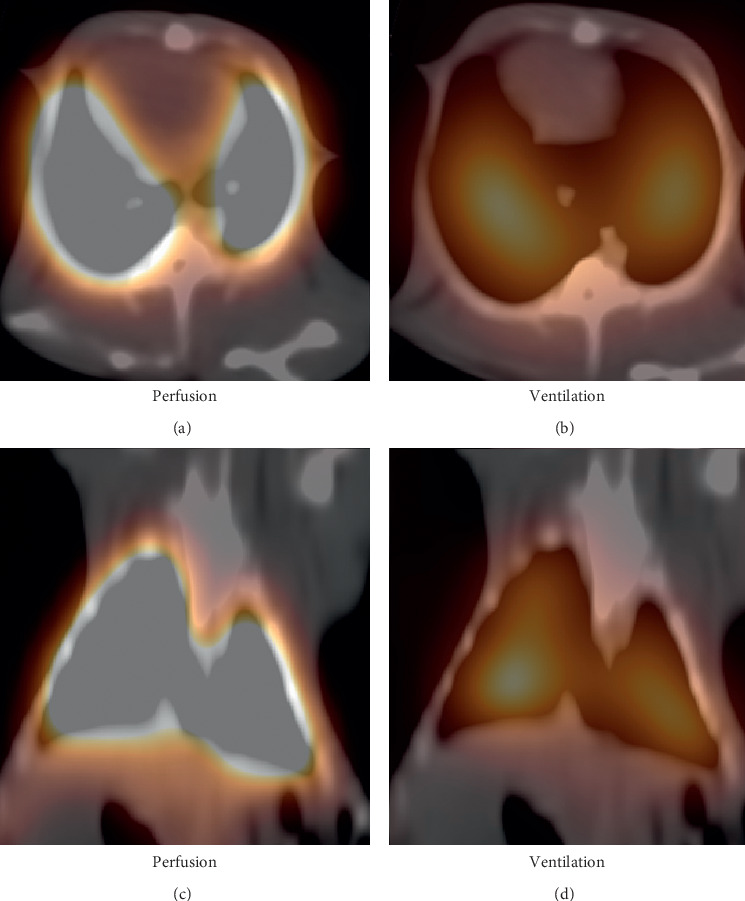
^68^Ga-MAA perfusion PET/CT and Technegas ventilation SPECT/CT images of a rabbit from the sham group. (a and c) Perfusion and (b and d) ventilation appear normal in both lung lobes.

**Figure 5:**
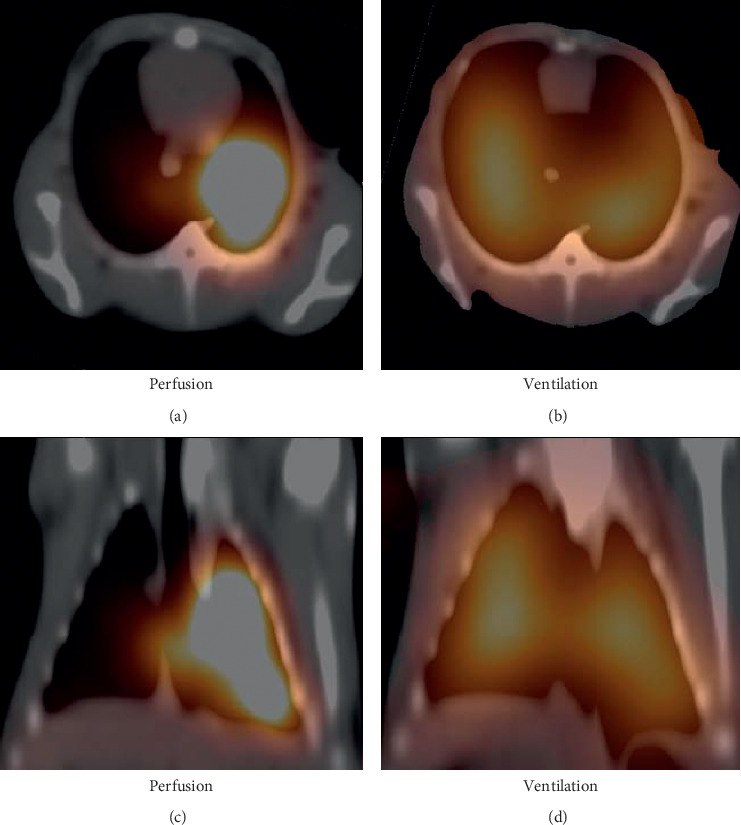
^68^Ga-MAA perfusion PET/CT and Technegas ventilation SPECT/CT images of a PE rabbit (PE5). There was a perfusion defect in the entire right lung and a perfusion defect in the upper left lobe of the left lung (a and c). No ventilation disorders were observed in the ventilation SPECT/CT of the same rabbit (b and d) (mismatch defects).

The ^68^Ga-MAA perfusion PET/CT images of all rabbits in the PE group are shown in Figure 3. Large, wedge-shaped perfusion defects were clearly observed in all cases. Ventilation SPECT/CT in these cases was normal. An example V/Q scan of one of the sham group rabbits is shown in Figure 4. The perfusion and ventilation of the lungs were completely normal. The V/Q scan findings of one of the rabbits in the PE group (PE5) are shown in Figure 5. A perfusion defect in the entire right lung and perfusion defect in one lobe in the left lung were observed. The ventilation SPECT/CT of this rabbit was completely normal. PE was diagnosed due to mismatch V/Q defects.

**Figure 6:**
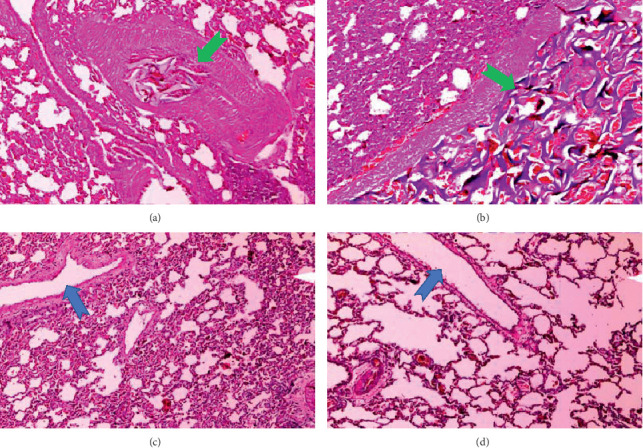
The sponge structure in the vessel filled with erythrocytes, bleeding in the surrounding parenchyma, and mononuclear cell infiltration (a and b; green arrows); healthy lung parenchyma and open vessel structures (c and d; blue arrows) (×10 H&E).

In the histopathological evaluation, intra-arterial gelatin sponge plugs, alveolar congestion, an increase in the number of monocytes in the parenchyma, and scattered emphysema areas were observed in the PE rabbits. In the histopathological evaluation of the lung tissues of the sham group rabbits, normal lung tissue was observed. No intra-arterial sponge plug was found (Figure 6). Sponge plugs were observed in 20 of 60 lung lobes of 12 rabbits that were examined histopathologically. Under macroscopic examination, the surfaces of the lungs with emboli were purple, and the normal lungs were pink.

With reference to the histopathological findings, the sensitivity, specificity, and accuracy of the V/Q scans are shown in Table 3.

## 4. Discussion

We synthesized ^68^Ga-MAA in an automated synthesis unit using a ^68^Ge/^68^Ga generator used for oncological studies and a commercial MAA kit at our institution. Our method was developed by modifying an existing method. The binding activity and radiochemical purity of our method was compatible with similar studies [14–16]. Our method is simple and fast because it is an automatic and closed system. In addition, the risk of radioactive and microbiological contamination is extremely low.

With reference to the histopathological results, there were 20 true-positive and 40 true-negative results from the V/Q scan measurements of the 60 lung lobes of the 12 rabbits evaluated. There were no false-positive or false-negative results. According to these results, when histopathological results are used as a reference, the sensitivity, specificity, and accuracy of V/Q scans were found to be 100%, 100%, and 100%, respectively. In a similar preclinical study with rabbits, Yang et al. [11] investigated the effectiveness of ^99m^Tc-MAA planar, SPECT, SPECT/CT perfusion, and pulmonary enhancement imaging (PEI) methods in PE. In this study, the sensitivity, specificity, and accuracy of ^99m^Tc-MAA planar, SPECT, and SPECT/CT perfusion images were found to be 71.0%, 84.4%, and 82.1%; 77.4%, 90.6%, and 86.3%; and 74.2%, 93.8%, and 87.4%, respectively. PEI is a method derived from dual-energy computed tomography (DECT). The sensitivity, specificity, and accuracy of PEI were 100%, 96.9%, and 97.9%, respectively, and were similar to our results. Specifically, high radiation exposure, renal dysfunction, and contrast allergy are the most important limitations of PEI. Our results are better than those from all conventional nuclear medicine methods for PE. The most important reason for this finding is that Yang et al. [11] decided on the presence of PE only according to a perfusion study. They preferred the PISAPED criteria as the evaluation method. In our study, we evaluated lung ventilation and perfusion studies together. Although scintigraphy and PET reflect similar physiological processes in evaluating lung perfusion, PET/CT has greater sensitivity, spatial and temporal resolution, and quantitative capacity than scintigraphy [17].

In clinical studies, the sensitivity of ^99m^Tc-V/Q SPECT/CT ranges from 80% to 100%, and its specificity ranges from 93% to 100% for PE [18]. ^99m^Tc-V/Q SPECT/CT has a high sensitivity and specificity. However, it is important that ^68^Ga-PET/BT V/Q imaging is now available as an alternative imaging method when ^99m^Tc supply crises are frequent. V/Q scanning is a vital imaging test. Due to its current technological advantages, it may be possible to use PET/CT more frequently in the future and perhaps use PET/CT instead of scintigraphy for the diagnosis of PE. Two clinical lung perfusion studies have been performed with ^68^Ga MAA. Mueller et al. [15] and Hofman et al. [19] stated that ^68^Ga-PET/CT V/Q scanning is superior to ^99m^Tc-SPECT/CT V/Q scanning in a pilot study with a few patients. However, they did not provide quantitative information (about sensitivity, specificity, and accuracy) due to the limited number of patients. According to the results of these studies [15, 19], ^68^Ga-MAA is a potential radiopharmaceutical for lung perfusion studies. The same research group has recently published new results. Le Roux et al. [20] (PECAN study) conducted a study on 24 patients and compared ^68^Ga V/Q scan and CTPA. They reported that ^68^Ga V/Q-PET/CT has potential benefits in patients with subsegmental embolism, in suspected PE cases, and in patients where CTPA is contraindicated.

In our study, we used Technegas for ventilation due to our technical conditions. Our method was applied for the first time in the V/Q scan. In approximately an hour, both ventilation and perfusion studies were completed. The disadvantages of our method are the difficulties in molybdenum supply and the difficulty of imaging in two separate devices. For these reasons, it is more convenient to do ventilation with Galligas in PET/CT. Previously, ventilation studies have been published with Galligas [21–23].

Our study has some limitations. First, the experimental rabbits did not have any lung disease. However, an accompanying lung disease is present in most real PE cases. It is, therefore, difficult to translate our results directly to clinical practice. Second, the short half-life of ^68^Ga-MAA poses a problem in adjusting the number of MAA particles to be delivered to the rabbits. This problem is not important when ^68^Ga-MAA is prepared as a unit dose for a single patient. Third, the perfusion defects were quite large in our cases, whereas smaller and peripheral defects can cause problems in the evaluation and change the results. Lastly, our results are limited by the small sample size. It may be possible to achieve significant results with a larger sample size and in human cases.

## 5. Conclusion

^68^Ga-MAA perfusion PET/CT scans combined with Technegas ventilation SPECT/CT have good sensitivity, specificity, and accuracy in the detection of PE in this animal model study.

## Figures and Tables

**Table 1:** ^68^Ga-MAA perfusion PET/CT, Technegas ventilation SPECT/CT, and histopathological results of all rabbits.

Rabbits	^68^Ga-MAA PET/CT (perfusion defect ±)					Technegas SPECT/CT (ventilation defect ±)					V/Q scan mismatch defect	Diagnosis (PE: ±)	
	^RU^	^RM^	^RL^	^LU^	^LL^	^RU^	^RM^	^RL^	^LU^	^LL^		V/Q scan	HP
PE 1	+	+	+	−	−	−	−	−	−	−	3	+	+
PE 2	+	+	+	+	−	−	−	−	−	−	4	+	+
PE 3	−	−	−	+	+	−	−	−	−	−	2	+	+
PE 4	−	−	+	+	+	−	−	−	−	−	3	+	+
PE 5	+	+	+	+	−	−	−	−	−	−	4	+	+
PE 6	+	+	+	+	−	−	−	−	−	−	4	+	+
Sham 1–6	−	−	−	−	−	−	−	−	−	−	0	**−**	**−**
Total number of lung lobes with mismatch defect in V/Q scan											20		

PE: pulmonary embolism; V/Q: ventilation and perfusion; HP: histopathologic; RU: right upper lobe; RM; right middle lobe; RL: right lower lobe; LU: left upper lobe; LL: left lower lobe.

**Table 2:** Comparison of V/Q scanning and HP findings.

Rabbits	^68^Ga-MAA PET/CT-Technegas SPECT/CT (mismatch defect)					HP results (location and size of PE)				
	RU	RM	RL	LU	LL	RU	RM	RL	LU	LL
PE 1	L	L	L	—	—	L	L	L	—	—
PE 2	L	L	L	L	—	L	L	L	L	—
PE 3	—	—	—	L	L	—	—	—	L	L
PE 4	—	—	L	L	L	—	—	L	L	L
PE 5	L	L	L	L	—	L	L	L	L	—
PE 6	L	L	L	L	—	L	L	L	L	—
Sham 1–6	—	—	—	—	—	—	—	—	—	—

PE: pulmonary embolism; V/Q: ventilation and perfusion; HP: histopathologic; RU: right upper lobe; RM; right middle lobe; RL: right lower lobe; LU: left upper lobe; LL: left lower lobe; L: lobe.

**Table 3:** Sensitivity, specificity, and accuracy values for combined^68^Ga-MAA PET/CT-Technegas SPECT/CT V/Q scans.

Statistics	Results (%)	CI (95%)
Sensitivity	100	83.16–100
Specificity	100	91.19–100
Accuracy	100	94.04–100

CI: confidence interval.

## Data Availability

The data used to support the findings of this study are available from the corresponding author upon request.
